# Catalpol Ameliorates Insulin Sensitivity and Mitochondrial Respiration in Skeletal Muscle of Type-2 Diabetic Mice Through Insulin Signaling Pathway and AMPK/SIRT1/PGC-1α/PPAR-γ Activation

**DOI:** 10.3390/biom10101360

**Published:** 2020-09-24

**Authors:** Kah Heng Yap, Gan Sook Yee, Mayuren Candasamy, Swee Ching Tan, Shadab Md, Abu Bakar Abdul Majeed, Subrat Kumar Bhattamisra

**Affiliations:** 1 School of Postgraduate Studies, International Medical University, Bukit Jalil, Kuala Lumpur 57000, Malaysia; yap.kahheng@student.imu.edu.my (K.H.Y.); TanSweeChing@student.imu.edu.my (S.C.T.); 2 Department of Life Sciences, School of Pharmacy, International Medical University, Bukit Jalil, Kuala Lumpur 57000, Malaysia; sookyee_gan@imu.edu.my (G.S.Y.); MayurenCandasamy@imu.edu.my (M.C.); 3 Department of Pharmaceutics, Faculty of Pharmacy, King Abdulaziz University, Jeddah 21589, Saudi Arabia; shaque@kau.edu.sa; 4 Universiti Teknologi MARA, Sungai Buloh-Selayang Medical-Dental Campus, Jalan Hospital, Sungai Buloh, Selangor 47000, Malaysia; abubakar@uitm.edu.my

**Keywords:** catalpol, type-2 diabetes mellitus, insulin sensitivity, glucose homeostasis, oxygen consumption rate, mitochondrial respiration, insulin signaling pathway

## Abstract

Catalpol was tested for various disorders including diabetes mellitus. Numerous molecular mechanisms have emerged supporting its biological effects but with little information towards its insulin sensitizing effect. In this study, we have investigated its effect on skeletal muscle mitochondrial respiration and insulin signaling pathway. Type-2 diabetes (T2DM) was induced in male C57BL/6 by a high fat diet (60% Kcal) and streptozotocin (50 mg/kg, i.p.). Diabetic mice were orally administered with catalpol (100 and 200 mg/kg), metformin (200 mg/kg), and saline for four weeks. Fasting blood glucose (FBG), HbA1c, plasma insulin, oral glucose tolerance test (OGTT), insulin tolerance test (ITT), oxygen consumption rate, gene (IRS-1, Akt, PI3k, AMPK, GLUT4, and PGC-1α) and protein (AMPK, GLUT4, and PPAR-γ) expression in muscle were measured. Catalpol (200 mg/kg) significantly (*p* < 0.05) reduced the FBG, HbA1C, HOMA_IR index, and AUC of OGTT whereas, improved the ITT slope. Gene (IRS-1, Akt, PI3k, GLUT4, AMPK, and PGC-1α) and protein (AMPK, p-AMPK, PPAR-γ and GLUT4) expressions, as well as augmented state-3 respiration, oxygen consumption rate, and citrate synthase activity in muscle was observed in catalpol treated mice. The antidiabetic activity of catalpol is credited with a marked improvement in insulin sensitivity and mitochondrial respiration through the insulin signaling pathway and AMPK/SIRT1/PGC-1α/PPAR-γ activation in the skeletal muscle of T2DM mice.

## 1. Introduction

Diabetes mellitus (DM) was the seventh leading cause of death in 2016 and 1.6 million people died due to DM globally [1]. Type 2 Diabetes mellitus (T2DM) is a metabolic disorder due to impaired insulin action and secretion. Being the greatest danger to public health in the 21st century, efforts required to prevent and control this disease are highly essential. Improvement in insulin sensitivity in T2DM patients is an important aspect of treatment as insulin resistance is generally undiagnosed and the body compensates by secreting excess insulin to keep the blood glucose within the normal value. The importance of insulin resistance in the etiology of diabetes mellitus has been well established [2]. According to the Whitehall II study, insulin sensitivity was already reduced 13 years before the onset of diabetes and the decline is steeper in the last five years before diagnosis. During the 13-year observation period, insulin secretion was elevated and showed a marked increase in the last 3–4 years before diagnosis, which is followed by a steep decline in insulin level [3]. Multiple mechanisms have been proposed for reduced insulin sensitivity in the liver, skeletal muscles, and adipose tissues. Skeletal muscle plays an important role in the pathogenesis of insulin resistance as it is responsible for the major part (>80%) of insulin-stimulated whole body glucose disposal [4]. The mechanisms for resistance in skeletal muscle include intracellular lipid accumulation, endoplasmic reticulum stress, impaired gene transcription, pro-inflammatory signals, and mitochondrial defects [5]. Mitochondrial dysfunction results in the intracellular accumulation of fatty acid metabolites, diacylglycerol (DAG), and long-chain fatty acyl-CoA (LCFA-CoA) [6]. DAG, LCFA-CoA, and ceramides activates Protein Kinase-C (PKC), which causes serine phosphorylation of insulin receptor substrate (IRS) proteins, leading to the inhibition of insulin signaling and development of insulin resistance [6,7,8]. Another potential mechanism that is responsible for insulin resistance is inflammation. Cytokines (tumor necrosis factor- α, interleukin-1β, and interleukin-6) and FFAs stimulate Toll-like receptor-mediated inflammatory signaling, which activates IκB kinase (IKK)-β and c-Jun N-terminal kinase (JNK). IKK-β and JNK are notable serine kinases that phosphorylate IRS-1 at serine residues thus decrease the insulin signaling. [9]. Mitochondrial dysfunction and consequent increases in ROS activate various serine kinases and inhibitor of nuclear factor kappa-B kinase subunit beta (IKKβ), which phosphorylates IRS proteins, leading to insulin resistance [10,11]. Thus, compounds that improve mitochondrial function which further augment insulin signaling pathway in skeletal muscle could be a potential therapy in the treatment of diabetes mellitus. Traditional medicines have great potential in the management of diabetes mellitus and the identification of active constituents that could enhance mitochondrial function and insulin signaling in skeletal muscle is an opportunity for the scientific community.

Catalpol, an iridoid glucoside obtained from the roots of *Rehmannia glutinosa*. This plant is traditionally used in China and Korea for the treatment of aging-related diseases and is extensively referred to as Di-Huang in traditional Chinese medicine for treating diabetic disorders [12]. Catalpol exerts a wide variety of biological activities including analgesic, sedative, liver protective, purgative, anti-inflammatory, anti-microbial, anti-tumour, and anti-apoptosis activity [13,14]. In the last few years, catalpol has been extensively investigated and several studies have reported its multiple biological activities. The antioxidant and free radical scavenging activity of catalpol are the key mechanisms for exhibiting neuroprotection, anti-atherosclerosis, cardioprotective, and antidiabetic activity [13]. The anti-hyperglycemic effect of catalpol was first investigated by Kitagawa and colleagues [14,15]. Hypoglycaemic activity of catalpol in high fat fed/STZ induced diabetic mice was suggested to have been mediated through improved muscle mitochondrial function. The study has reported an increased mitochondrial ATP production, mtDNA copy number, mitochondrial membrane potential, and expression of peroxisome proliferator-activated receptor gamma co-activator 1 (PGC1) α mRNA [16]. In another study, catalpol demonstrated an antidiabetic effect in db/db mice, which is associated with activation of the phosphoinositide 3-kinases (PI3K)/protein kinase B (Akt) pathway and augmented skeletal muscle myogenesis [17]. The same group of researchers reported that catalpol activated AMPK/PGC-1α/TFAM signaling, which augments mitochondrial biogenesis in skeletal muscle, thereby increasing glucose uptake and ATP production [18]. In db/db mice, catalpol showed improvement in glucose tolerance and reduced insulin resistance. The antidiabetic activity was linked to increased glucose transporter (GLUT)-4 protein expression in skeletal muscle and adipose tissue; reduced expression of Acetyl-CoA carboxylase (ACC) and 3-hydroxy-3-methyl-glutaryl-coenzyme A reductase (HMGCR) in the liver [19]. Catalpol ameliorated hepatic insulin resistance and reduced diabetes-associated hepatic injury and steatosis in HFD/STZ induced diabetic mice which was mediated through activation of 5′ adenosine monophosphate-activated protein kinase (AMPK), increased glycogen synthase kinase 3 beta (GSK3β) phosphorylation, reduced glycogen synthase phosphorylation, stimulation of hepatic glycogen synthesis, and inhibition of hepatic gluconeogenesis [20]. Further, the antidiabetic effect of catalpol in HFD fed mice was linked to reduced inflammation. It was reported that catalpol showed a reduction in macrophage infiltration into adipose tissue, reduced pro-inflammatory cytokines (TNF-α, IL-6, and IL-1β), and increased anti-inflammatory marker (IL-10) expression in adipose tissue. Reduced phosphorylation of IKKβ and JNK and reduced NF-kB p50 activation in adipose tissue from catalpol-treated mice suggested that the insulin-sensitizing effect of catalpol may be due to the attenuation of inflammation in adipose tissue through JNK and NF-kB signaling pathways [21]. In our recent study, we have reported that catalpol at (200 mg/kg, p.o.) showed a significant reduction in fasting blood glucose (FBG), homeostatic model assessment for insulin resistance (HOMA_IR), plasma, and liver triglyceride. The effect of catalpol was correlated with the increased Peroxisome proliferator-activated receptor gamma (PPAR-γ) gene and protein expression, glucokinase gene expression in the liver tissue further, the glycogen content in hepatocyte were reversed by catalpol in T2DM mice. This study suggested the role of catalpol in PPAR-γ expression that improves insulin sensitivity in the liver [22]. Further, catalpol have demonstrated an increased insulin sensitivity through activation of the insulin signaling pathway in pancreatic beta-cells (INS-1E) [23]. In this study, we have investigated the antidiabetic effect of catalpol in high fat diet/multiple low dose STZ induced type 2 diabetic mice. Further, catalpol’s effect is corroborated with the improved mitochondrial respiration and insulin signaling pathway in the skeletal muscle of type-2 diabetic mice.

## 2. Materials and Methods

### 2.1. Drugs and Reagents

Catalpol (≥98% purity) was obtained from Shanghai PI Chemicals Ltd., Shanghai, China. Metformin hydrochloride was obtained as a gift sample from Pharmaniaga manufacturing Berhad., Malaysia. Streptozotocin (STZ) (Santa Cruz Biotechnology, Inc., Dallas, TX, USA), insulin (Novo Nordisk Ltd., Crawley, UK), and all other reagents were obtained from Sigma-Aldrich (St. Louis, MO, USA).

### 2.2. Animal Studies

Five to six-week-old male C57BL/6N mice were acquired from Brain Research Institute Monash Sunway (BRIMS), Monash University, Malaysia and were housed in individual cages in Animal Holding Facilities, International Medical University, Kuala Lumpur, Malaysia. The animals were randomly housed in polypropylene cages under standard conditions of dark and light cycle (12/12 h), humidity (50–60%), and temperature (20–24 °C) maintained at the animal house facility of International Medical University, Bukit Jalil, Kuala Lumpur, Malaysia. All the animals received humane care and had access to food and water ad libitum. The experiments were performed according to the criteria outlined in the “Guide for the care and use of Laboratory Animals” and the protocol obtained prior approval from IMU Joint-committee on research & ethics, International Medical University (Approval number: 4.4/JCM-121/2016).

Upon arrival, the animals underwent one week of acclimatization during which all animals were fed with normal chow pellets (12% Kcal energy as fat) (Specialty Feeds, Glen Forrest, Australia). During the subsequent week, the animals were randomised according to their body weight and one-fifth of the animals were allocated to the normal control group where they would be fed with normal chow pellets throughout the study. Whereas the remaining animals were fed with high fat diet (HFD) (60% Kcal energy as fat) (Altromin C1090-60, Altromin GmbH, Lage, Germany) for two weeks to induce insulin resistance; the diet was continued to the end of the study. Subsequently, T2DM was induced in the animals by administering STZ (50 mg/kg, i.p.) for three consecutive days while normal control animals received equal volume of citrate buffer. The animals were allowed to develop T2DM for one week. The animals having FBG ≥ 200 mg/dL were selected for the study. The groupings of animals were group 1: normal control mice treated with saline; group 2: diabetic control mice treated with saline; group 3: diabetic mice treated with catalpol (100 mg/kg, p.o.); group 4: diabetic mice treated with catalpol (200 mg/kg, p.o.), and group 5: diabetic mice treated with metformin (200 mg/kg, p.o.). All the treatments were carried out for four consecutive weeks. Till the completion of study, group-1 was fed with normal chow pellet whereas, group-2 to group-5 were fed with a high fat diet. All the animals were sacrificed at the end of the study. Gastrocnemius and soleus muscle were isolated and fresh muscle tissues were used for the determination of mitochondrial respiration and oxygen consumption rate. A portion of muscle was immediately snap-frozen in liquid nitrogen and was stored at −80 °C until the measurement of gene and protein expression.

### 2.3. Measurement of FBG, HbA1C, Plasma Insulin, HOMA_IR

Fasting blood glucose and HbA1C was measured in overnight fasted animals by puncturing the tail vein. Accu-Chek Active glucometer (Roche Diagnostics GmbH, Basel, Switzerland) and A1CNow^®^+ Professional (PTS Diagnostics, Whitestown, IN, USA) were used respectively for the measurement of FBG and HbA1C [24,25]. Blood was collected from retro-orbital sinus puncture and centrifuged to collect the plasma for estimation of insulin using a mouse insulin ELISA kit (Millipore, Burlington, MA, USA). Insulin resistance was determined using the homeostatic model assessment of insulin resistance (HOMA_IR) equation. The product of fasting blood glucose (mmol/L) and insulin (mU/mL) is divided by a constant of 22.5. Greater insulin resistance is represented by a higher HOMA_IR score [26].

HOMA_IR = {Fasting insulin level (mU/mL) × Fasting glucose (mmol/L)}/22.5

### 2.4. Oral Glucose Tolerance Test (OGTT) and Insulin Tolerance Test (ITT)

OGTT and ITT were performed upon completion of the four-week dosing regimen. OGTT was performed in overnight fasted mice on Day 28. The animals received D-glucose (2.0 g/kg) by oral gavage. Blood glucose levels were measured before the glucose load and at 30, 60, 90, and 120 min after the glucose administration. ITT was performed in 4 h fasted mice on Day 30. The animals were administered insulin (0.75 U/kg, i.p.). Blood glucose levels were measured before and at 15, 30, 60, 90, and 120 min after the insulin administration [26].

### 2.5. Gene Expression Study

Total RNA was isolated from the skeletal muscle of the mice using RNeasy^®^ Plus Universal Mini Kit (Qiagen, Hilden, Germany). Approximately 30 mg of skeletal muscle was for total RNA isolation. Total isolated RNA (2.5 µg) was reverse-transcribed to synthesize cDNA using QuantiNova Reverse Transcription Kit (Qiagen, Hilden, Germany) for each sample and diluted to 20 ng/µL before qPCR. The resultant cDNA samples were used immediately for quantitative real-time PCR or transferred to −20 °C freezer for long term storage. The cDNA was used as the template to amplify the target genes using RT-qPCR in a 20 µL total volume of 5 µL cDNA (40 ng), 10 µL QuantiNova SYBR Green (Qiagen, Hilden, Germany), 2 µL QuantiTect Primers (Qiagen, Hilden, Germany) and 3 µL RNase-free water. RT-qPCR was performed using CFX96TM Real-Time PCR Detection System (Bio-Rad Laboratories, Inc., Philadelphia, PA, USA). The following cycling conditions were used: PCR initial activation step at 95 °C for 2 min then 40 cycles of denaturation step at 95 °C for 15 sec followed by extension step at 60 °C for 60 sec. All gene expression levels were normalized to GAPDH. The delta-delta T (2^−ΔΔCT^) method was used to calculate relative expression values. The QuantiTect Primers from Qiagen (Hilden, Germany) used are Gapdh (Mus musculus; QT01658692); Irs1 (Mus musculus; QT00251657); Akt2 (Mus musculus; QT00136969); Pik3r1 (Mus musculus; QT01543934); Slc2a4 (Mus musculus; QT01044946); Prkaa1 (Mus musculus; QT00286923); Sirt 1 (Mus musculus; QT01546083); Ppargc1a (Mus musculus; QT02524242).

### 2.6. Protein Expression Study

Tissues (~20 mg of skeletal muscle) were lysed in 900 μL RIPA buffer (Nacalai Tesque, Inc., Kyoto, Japan) supplemented with protease inhibitor and phosphatase inhibitor (Nacalai Tesque, Inc., Kyoto, Japan). The protein samples were diluted to 1 mg/mL with ultrapure water and Laemmli Buffer (lysate to Laemmli Buffer 1:1 ratio). The samples were heated at 95 °C for 5 min to denature the proteins prior to Western Blot. A total of 30 μg protein sample was separated by SDS-PAGE gel (4% stacking + 7.5% resolving) and transferred onto a polyvinylidene difluoride (PVDF) membrane (Merck Millipore, Burlington, MA, USA) using Mini-PROTEAN Tetra Cell (Bio-Rad, Hercules, CA, USA). After blocking with Blocking One Solution (Nacalai Tesque, Kyoto, Japan) for 1 h at room temperature, the membranes were incubated with primary antibody overnight at 4 °C. On the next day, the membranes were washed with TBST three times for 5 min each. Subsequently, the membranes were incubated with the corresponding HRP-labelled secondary antibody at room temperature for 1 h followed by three-times washing with TBST for 5 min each. The immune complex was detected using the Chemi-Lumi One-L or Chemi-Lumi One Super (Nacalai Tesque, Kyoto, Japan). Protein bands were visualized with Molecular Imager ChemiDocTM XRS+ System (Bio-Rad, Hercules, CA, USA) and the intensities were analysed with Image Lab software (Bio-Rad, Hercules, CA, USA). All the values were normalized with β-actin which is used as the endogenous control. All the primary and secondary antibodies used were obtained from Cell Signaling Technology, USA. The list of antibodies includes phospho-AMPKα (Thr172) (Rabbit mAb; dilution: 1:1000; Cat #2535); AMPKα (Rabbit mAb; dilution: 1:1000; Cat #5832); GLUT4 (Mouse mAb; dilution: 1:1000; Cat #2213); β-Actin (Rabbit mAb; dilution: 1:1000; Cat #4970); PPAR-γ (Rabbit mAb; dilution: 1:1000; Cat #2443); horseradish peroxidase-conjugated secondary anti-rabbit (dilution: 1:3000; Cat #7074), and anti-mouse (dilution: 1:3000; Cat #7076) antibodies.

### 2.7. Mitochondria Function Study

Mitochondria were isolated from the fresh skeletal muscle tissues using MitoCheck^®^ mitochondria isolation kit (Cayman Chemical, Ann Arbor, MI, USA) according to the manufacturer’s protocol with some modifications. The mitochondrial protein concentration was determined using protein assay BCA Kit (Nacalai Tesque, Kyoto, Japan). The mitochondrial suspension was diluted to obtain 1 mg/mL of protein prior to subsequent assay. Oxygen consumption rate was measured using MitoCheck^®^ Mitochondrial oxygen consumption rate (OCR) assay kit (Cayman Chemical, Ann Arbor, MI, USA) according to the manufacturer’s protocol. This assay kit utilises a phosphorescent oxygen probe called MitoXpress^®^-Xtra whose signal increases as the oxygen concentration decreases. Firstly, 110 μL state 3 respiration buffer containing diluted mitochondrial samples was added to sample wells whereas 110 μL state 3 respiration buffer alone was added to background signal wells on a 96-well black clear bottom polystyrene plate. Then 10 μL MitoXpress^®^-Xtra was added to all wells. Following this, 20 μL test compounds (mitochondrial respiration buffer for state 3 respiration, 10 ng/mL oligomycin for state 4 respiration or 10 μg/mL antimycin A for full inhibition) were added to the designated wells. The assay was initialized by adding 20 μL succinate (80 mM) to all wells prior to the addition of 100 μL of pre-warmed mineral oil to seal the reaction. Immediately the plate was read kinetically at 37 °C for 30 min with SpectraMax M3 plate reader using excitation wavelength of 380 nm and emission wavelength of 650 nm. From the plot of MitoXpress^®^-Xtra signal versus time (minutes) of each sample, the linear portion of the signal profile was selected and linear regression was applied to determine the slope for each of the signal profiles. Appropriate average and standard deviation of the slope values for sample replicates were calculated. The slope obtained for the background wells was subtracted from all test values. The final slope value equals the rate of oxygen consumption in the sample.

### 2.8. Citrate Synthase (CS) Activity

Citrate synthase (CS) activity is used as the biomarker for the mitochondrial content in tissue homogenate. Skeletal muscle isolated from the mice were used immediately for the CS assay. The skeletal muscle of six animals was from each group pooled together for the assay. Pooled skeletal muscles were homogenized using tissue ruptor (Qiagen, Hilden, Germany) in lysis buffer (10 mM Tris, pH7.4, 250 mM sucrose and 1 mM EDTA) followed by leaving on ice for 30 min. The lysates were then centrifuged at 13,000 rpm at 4 °C for 30 min. Samples containing 45 μg of total protein were used to determine the CS activity via a plate-based colorimetric kit, MitoCheck^®^ Citrate synthase activity assay (Cayman Chemical, Ann Arbor, MI, USA). The kit protocol was followed for the measurement of CS in the samples. This enzyme activity was determined in kinetic assay measuring the absorbance at 412 nm. The corrected absorbance was calculated by subtracting the absorbance of 0 min (before the reaction) and the graph was plotted. The slope was calculated from the linear part of the graph which lies within the initial 3 min of the assay. CS activity was calculated using the slope value in the formula provided in the kit catalogue. Each sample is measured in triplicate from each group. The CS activity was expressed as µmoles/min/mg protein.

### 2.9. Statistical Analysis

Results were expressed as mean ± standard error of mean (SEM). The statistical package SPSS version 18 was used to analyse the data. One-way analysis of variance (ANOVA) followed by the post hoc Dunnett’s test was used for statistical comparison between the groups. *p* value of <0.05 was considered as statistically significant.

## 3. Results

### 3.1. Measurement of FBG, HbA1C, Plasma Insulin, HOMA_IR

**Figure 1:**
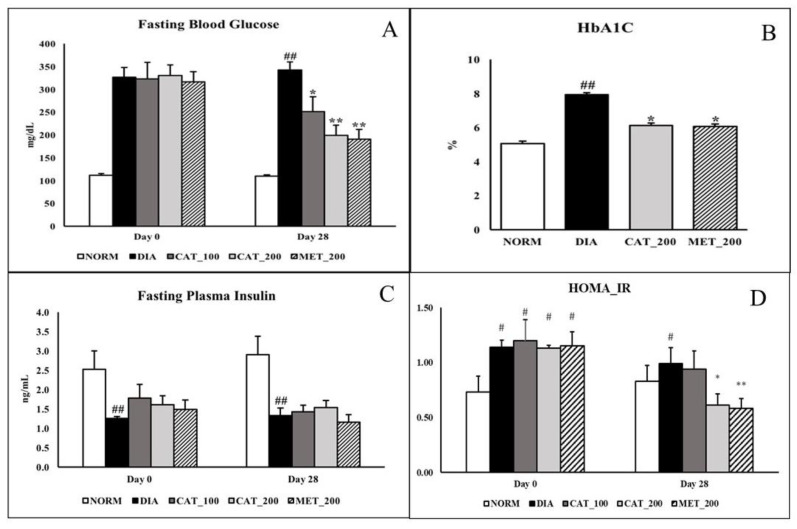
Effect of catalpol and metformin on FBG, plasma insulin, HbA1C and HOMA_IR index. (**A**) FBG at baseline (Day 0) and 28 days; (**B**) HbA1C on day 28; (**C**) Plasma insulin level; (**D**) HOMA_IR index. NORM: Normal control; DIA: Diabetic control; CAT_100: Catalpol (100 mg/kg, p.o.); CAT_200: Catalpol (200 mg/kg; p.o.); MET_200: Metformin (200 mg/kg, p.o.). Data were expressed in mean ± SEM (*n* = 8). # *p* < 0.05 and ## *p* < 0.01 vs. NORM; * *p* < 0.05 and ** *p* < 0.01 vs. DIA.

Fasting blood glucose was measured at baseline (day 0) and day 28 in overnight fasted animals. On day 28, the FBG of diabetic control animals was 342.38 ± 17.92 mg/dL. whereas catalpol (100 mg/kg)-treated mice showed significant (*p* < 0.05) reduction to 251.13 ± 32.98 mg/dL while the reduction was more significant (*p* < 0.01) in both the catalpol (200 mg/kg) and metformin (200 mg/kg)-treated mice with the FBG of 199.50 ± 21.72 mg/dL and 191.13 ± 21.25 mg/dL respectively (Figure 1A). As shown in Figure 1B, Normal control animals exhibited the HbA1C of 5.06 ± 0.13% which is significantly (*p* < 0.05) increased in diabetic control animals (7.92 ± 0.54%). Treatment with 200 mg/kg of catalpol and metformin significantly (*p* < 0.05) reduced HbA1C to 6.12 ± 0.31% and 6.07 ± 0.52% respectively. Plasma insulin concentration in normal control animals were 2.53 ± 0.37 and 3.36 ± 0.49 ng/mL on day 0 and day 28 respectively. In diabetic control group the insulin level was significantly (*p* < 0.05) low with 1.26 ± 0.04 and 1.34 ± 0.14 ng/mL on day 0 and 28 respectively. Catalpol and metformin treatment didn’t show any significant change in plasma insulin level on day 28 (Figure 1C). Normal animals recorded HOMA_IR value of 0.73 ± 0.14 and 0.83 ± 0.15 at day 0 and day 28 respectively. On day 28, HOMA_IR index in diabetic control group was significantly (*p* < 0.05) increased to 0.99 ± 0.15 whereas, intervention with catalpol (200 mg/kg) and metformin (200 mg/kg) significantly (*p* < 0.05) attenuated the index to 0.61 ± 0.10 and 0.58 ± 0.09 respectively (Figure 1D).

### 3.2. Oral Glucose Tolerance Test (OGTT) and Insulin Tolerance Test (ITT)

**Figure 2:**
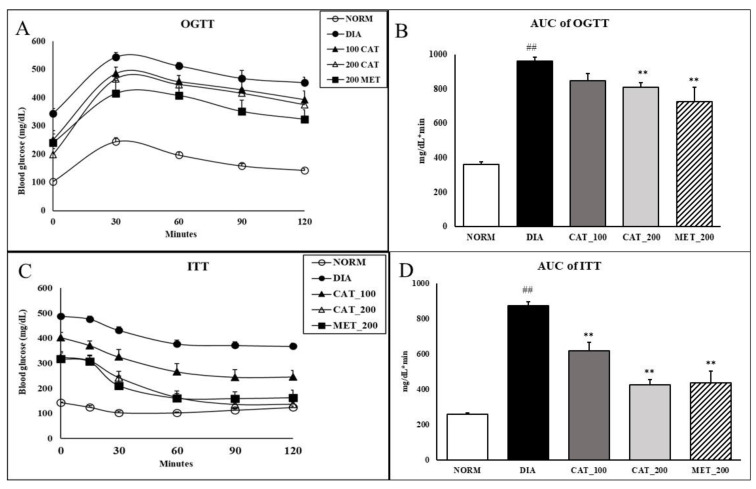
Effect of catalpol and metformin on OGTT and ITT on day 28 of treatment. (**A**) OGTT curve; (**B**) AUC of OGTT; (**C**) ITT curve; (**D**) AUC of ITT. NORM: Normal control; DIA: Diabetic control; CAT_100: Catalpol (100 mg/kg, p.o.); CAT_200: Catalpol (200 mg/kg; p.o.); MET_200: Metformin (200 mg/kg, p.o.). Data were expressed in mean ± SEM (*n* = 8). ## *p* < 0.01 vs. NORM; ** *p* < 0.01 vs. DIA.

Impaired glucose tolerance reflected in a larger incremental area under the curve (AUC) of the OGTT. The AUC of diabetic control was significantly (*p* < 0.01) higher than normal control animals. Animals treated with 200 mg/kg of catalpol and metformin exhibited significant (*p* < 0.01) improvement in glucose clearance as demonstrated by 18.3% and 17.2% reduction in the AUC compared with the diabetic control group. Catalpol (100 mg/kg) didn’t show any significant reduction in AUC of OGTT as compared to diabetic control (Figure 2A,B). In nondiabetic rats, blood glucose was markedly reduced up to 30% as compared to baseline value within 30 min and the blood glucose was elevated towards baseline value at 120 min. However, in diabetic control animals, the reduction in blood glucose was only 12% at 30 min which is further extended upto 120 min with 22% reduction. In catalpol (200 mg/kg) and metformin (200 mg/kg), the reduction in glucose at 30 min was 21% and 24% respectively which is further significantly (*p* < 0.01) extended upto 120 min with a reduction of 60% and 44% in blood glucose level. This indicates the glucose disposal by insulin sensitizing effect of catalpol and metformin. The AUC of ITT in diabetic control animals showed a significantly (*p* < 0.01) higher than normal control. Further, the AUC was significantly (*p* < 0.01) reduced in catalpol and metformin treated animals (Figure 2C,D).

### 3.3. Gene Expression Study

**Figure 3:**
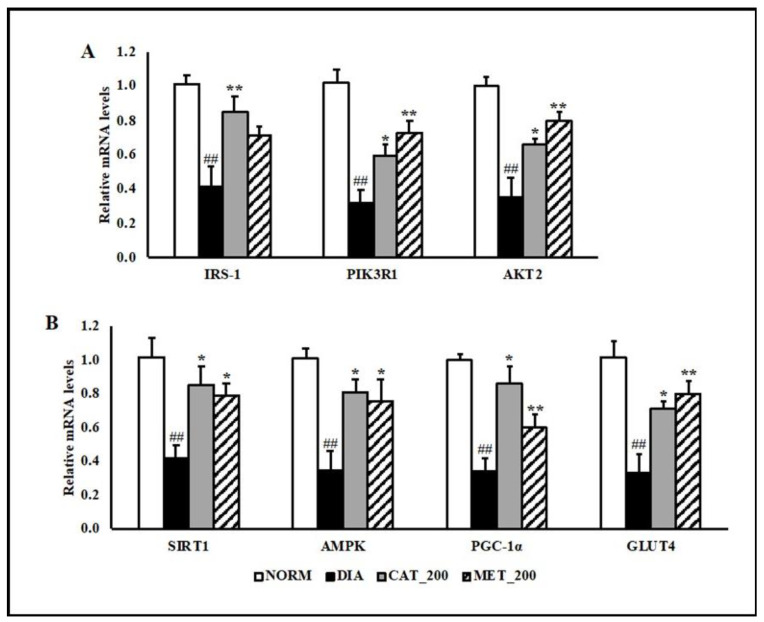
Effect of catalpol and metformin on expression of genes involved in insulin signaling pathways, AMPK, PGC-1α, SIRT1 and GLUT4 in skeletal muscle (gastrocnemius and soleus muscle) (**A**) genes involved in insulin-signaling pathway (**B**) AMPK-SIRT1-PGC-1α-GLUT4 pathway in skeletal muscles. NORM: Normal control; DIA: Diabetic control; CAT_200: Catalpol (200 mg/kg; p.o.); MET_200: Metformin (200 mg/kg, p.o.). Data represents mean ± SEM (*n* = 6–8). ## *p* < 0.01 vs. NORM; * *p* < 0.05 and ** *p* < 0.01 vs. DIA.

Insulin receptor substrate 1 (IRS-1), phosphatidylinositol 3-kinase regulatory subunit alpha (PIK3R1) and RAC-beta serine/threonine-protein kinase (Akt2) gene expression was determined in skeletal muscle of all the animals. The transcript expression of IRS-1, PIK3R1 and Akt2 were significantly (*p* < 0.01) down-regulated in the skeletal muscles of diabetic mice compared to normal control. Administration of catalpol (200 mg/kg) and metformin (200 mg/kg) reversed the reduction and their mRNA levels were significantly (*p* < 0.01) higher than diabetic control (Figure 3A). In addition to insulin signaling pathway, gene expression of 5′ adenosine monophosphate-activated protein kinase (AMPK) was investigated in order to validate the results with mitochondrial function study. Peroxisome proliferator-activated receptor gamma coactivator 1-alpha (PGC-1α), sirtuin 1 (SIRT1), AMPK, and glucose transporter type 4 (GLUT4) were significantly (*p* < 0.01) down-regulated in the diabetic skeletal muscles. Catalpol (200 mg/kg) and metformin (200 mg/kg) treated animals showed profound (*p* < 0.01) improvement in SIRT1, PGC-1α, AMPK, and GLUT4 expression in the skeletal muscle as compared to diabetic control. The transcript level of PGC-1α, which is co-regulated by SIRT1 and AMPK, were normalized by catalpol treatment and to a lesser extent in the metformin group. Consequently, skeletal muscle expression of GLUT4 was rescued in both the treatment groups (Figure 3B).

### 3.4. Protein Expression Study

**Figure 4:**
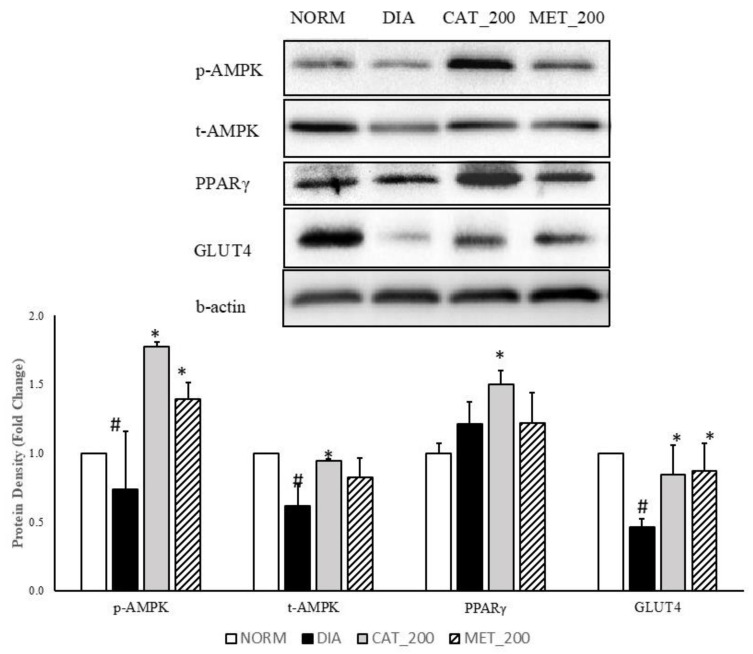
Effect of catalpol and metformin on proteins (AMPK, PPAR-γ and GLUT4) in skeletal muscle (gastrocnemius and soleus muscle). NORM: Normal control; DIA: Diabetic control; CAT_200: Catalpol (200 mg/kg; p.o.); MET_200: Metformin (200 mg/kg, p.o.). Data represents mean ± SEM (*n* = 6–8). # *p* < 0.05 vs. NORM and * *p* < 0.05 vs. DIA.

Total-AMPK, p-AMPK, PPAR-γ, and GLUT4 protein expression was determined in the skeletal muscle of all the animals. The expression of p-AMPK, total-AMPK and GLUT4 was significantly (*p* < 0.05) transcript expression of IRS-1, PIK3R1 and AKT2 were significantly (*p* < 0.01) down-regulated in the skeletal muscles of diabetic mice compared to normal control. p-AMPK, total AMPK, and GLUT4 protein was reduced with 26%, 39% and 54% respectively as compared to normal control. Administration of catalpol (200 mg/kg) and metformin (200 mg/kg) reversed the reduction of these proteins and significantly (*p* < 0.05) higher than diabetic control. Catalpol (200 mg/kg) has improved p-AMPK, total-AMPK and GLUT4 expression with 140%, 53% and 83% respectively and metformin with 88%, 34%, and 88%, respectively, as compared to diabetic control mice. The fold change of PPAR-γ protein expression in diabetic control was 1.21 ± 0.11 vs. normal control in the skeletal muscle. Higher expression level was found in diabetic control although the changes are not significant as compared to normal control. Catalpol treatment significantly (*p* < 0.05) increased the PPAR-γ expression (1.50 ± 0.07 fold) as compared to diabetic control. Metformin treatment showed no change in PPAR-γ expression (1.22 ± 0.16) as compared to diabetic control (Figure 4).

### 3.5. Mitochondria Function Study

**Figure 5:**
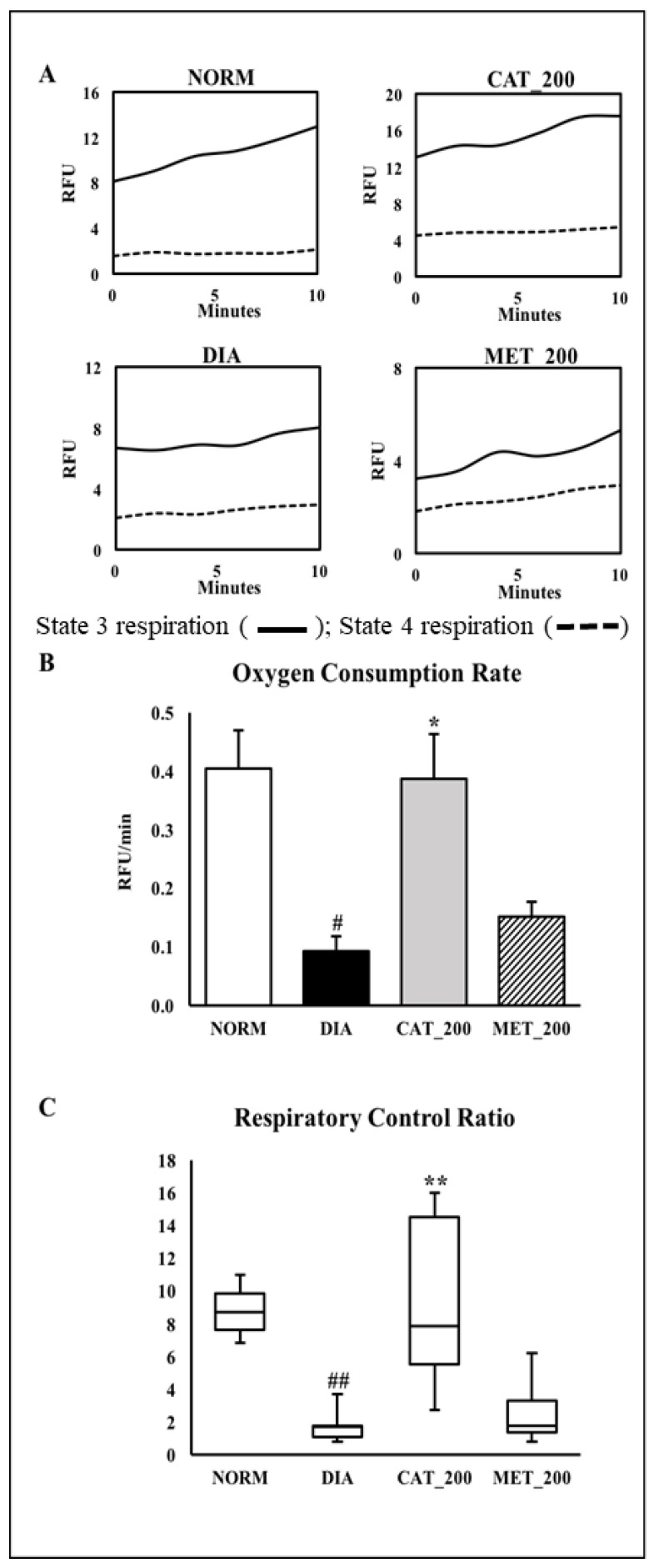
Effect of catalpol on state-3/stae-4 respiration, oxygen consumption rate (OCR) and respiratory exchange rate (RCR) (**A**) Representative trace of oxygen consumption shown by relative fluorescent units (RFU) over time between 0 and 10 min. (**B**) Oxygen onsumptionrate of isolated mitochondria from skeletal muscle. The slopes of the oxygen consumption curves were measured between 0 and 10 min; (**C**) Respiratory Control Ratio (RCR) was calculated as the ratio of State 3 versus State 4 respiration slope. NORM: Normal control; DIA: Diabetic control; CAT_200: Catalpol (200 mg/kg; p.o.); MET_200: Metformin (200 mg/kg, p.o.). Data represents mean ± SEM (*n* = 3 per group as pooled sample). # *p* < 0.05 and ## *p* < 0.01 vs. NORM; * *p* < 0.05 and ** *p* < 0.01 vs. DIA.

The main physiological function of mitochondria is the generation of ATP by oxidative phosphorylation. Hence, the assessment of oxygen consumption rate (OCR) was used to assess mitochondrial function. Oxygen consumption was measured in isolated mitochondria from mice skeletal muscles. State 4 respiration was measured in the presence of complex V inhibitor oligomycin. The difference in oxygen consumption rates in the absence and presence of oligomycin indicate as mitochondria were coupled. The skeletal muscle of normal animals showed a higher slope in state-3 respiration, indicating an increased conversion of ADP to ATP. This showed a normal mitochondrial function with a higher conversion of ADP to ATP. In diabetic animals, the slope of state 3 respiration was insignificant as compared to the normal control group which indicates a low conversion of ADP to ATP. However, Catalpol (200 mg/kg) showed a higher slope which supports a better mitochondrial function however, metformin didn’t improve the slope as compared to diabetic control (Figure 5A). Oxygen consumption rate (OCR) and respiratory control ratio (RCR) were significantly (*p* < 0.01) lowered in diabetic control animals as compared to the normal control group. Catalpol treatment exhibited a significant (*p* < 0.01) improvement in OCR and RCR, whereas a similar response was not observed in metformin treated group (Figure 5B,C).

### 3.6. Citrate Synthase (CS) Activity

**Figure 6:**
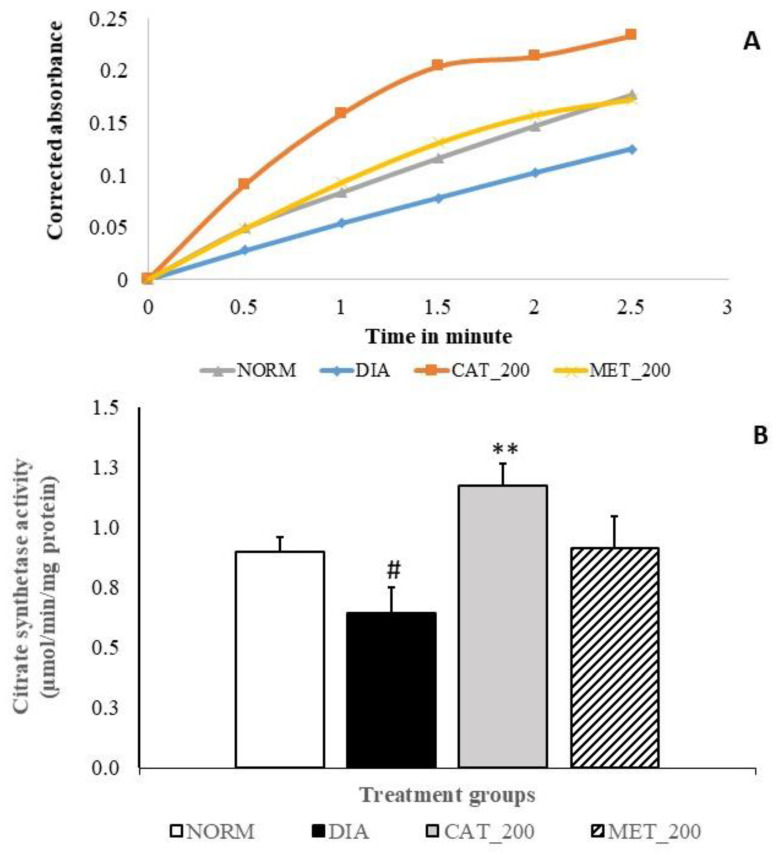
Effect of catalpol on CS activity in skeletal muscle (**A**) Slope of CS reaction (reaction rate) measured between 0 to 3 min (**B**) CS activity of different treatment groups. NORM: Normal control; DIA: Diabetic control; CAT_200: Catalpol (200 mg/kg; p.o.); MET_200: Metformin (200 mg/kg, p.o.). Data represents mean ± SEM (*n* = 3 per group as pooled sample). #*p* < 0.05 vs. NORM and **p < 0.01 vs. DIA.

Slope of CS assay indicates the reaction rate of the enzyme. Higher enzyme levels indicate greater slope. Slope of reaction in diabetes control and normal control are 0.05 and 0.07 respectively and diabetes control showed significant (28%; *p* < 0.05) in CS activity as compared to normal mice. Catalpol treatment showed significant (82%; *p* < 0.01) improvement CS activity as indicated in higher slope value (0.09) vs. diabetes control and 31% vs. normal control. In contrast, metformin treatment showed a higher reaction rate with a slope value of 0.07 as compared to the diabetes control, CS activity was 41% higher vs. diabetic control, and almost no change versus normal control. The results are depicted in Figure 6A,B.

## 4. Discussion

Type-2 diabetes mellitus is a complex metabolic disorder and simulating the complexity of this disorder in experimental animal models is remain a great challenge for the scientific community. We have used high fat diet (60% KCal) and multiple low dose STZ model in this study where HFD was administered initially for the first two weeks followed by low dose STZ injection for three consecutive days to induce the T2DM in C57BL/6 mice. Throughout the duration of four weeks of drug treatment, HFD was continued until the completion of the study. This animal model is reported to be a robust model that simulates the human condition. Feeding with 60% Kcal HFD produces hyperinsulinemia and insulin resistance initially followed by treatment with STZ that causes the beta-cell destruction resulting normal to low levels of insulin concentration in non-genetic, outbred animals such as mice [27,28]. In our experiment, we have observed that mice developed with T2DM have demonstrated a constant elevation of fasting blood glucose more than 300 mg/dl throughout the study without fluctuation in plasma insulin level which was 1.26 ± 0.04 ng/mL and 1.34 ± 0.14 ng/mL respectively on day 0 and day 28 of treatment. Further, the insulin resistant index i.e., HOMA_IR was significantly higher than the normal control animals. Blood HbA1C level in the diabetic control group was significantly higher than normal mice (7.92 ± 0.53 vs. 5.16 ± 0.13), which is in agreement with the reported HbA1C levels in diabetic mice [29]. The data in T2DM control animals remained consistent throughout the study duration suggesting a well validated model for testing antidiabetic drugs. Catalpol treatment at 200 mg/kg for four consecutive weeks to these animals significantly reduced the FBG, HOMA_IR and HbA1c levels. However, it didn’t exhibit any significant effect on plasma insulin concentration. Low dose catalpol (100 mg/kg) significantly reduced the FBG but didn’t show any effect on other glycaemic parameters suggesting its low efficacy at this dose. Metformin was used as the standard drug which also exhibited a similar effect on glycaemic parameters without altering the plasma insulin concentration. Thus, it suggests that catalpol has no effect on insulin secretion. Rather, it may attenuate the insulin resistance through which it exhibits its antidiabetic effect.

Other tests like OGTT and ITT were widely accepted to find out glucose tolerance and insulin sensitivity respectively. OGTT is a simple test used to diagnose glucose intolerance in clinical practice. OGTT reflects the efficiency of the body to dispose glucose after oral glucose load or meal. The OGTT or meal tolerance test mimics the glucose and insulin dynamics of physiological conditions more closely than conditions of the glucose clamp or insulin sensitivity test. Impaired glucose tolerance is reflected in a larger incremental AUC of the glucose disappearance curve [26]. Results of OGTT revealed that the AUC(_0–120 min_) of the glucose disappearance curve was significantly higher in diabetic control as compared to the normal group which reflects a greater glucose intolerance in diabetic control animals. The AUC was significantly attenuated in catalpol and metformin treatment thus, glucose intolerance is markedly reduced after the treatment of catalpol and metformin. ITT, a simple and easy to perform test than the glucose clamp study. The glucose disappearance rate/slope of glucose disposal after insulin administration has a close correlation with glucose clamp studies [30,31,32]. Insulin resistance is estimated by ITT and it could be a useful tool for predicting the effectiveness of insulin sensitizers [33]. The slope of the glucose disposal in ITT was significantly higher than the diabetic control by catalpol and metformin. A high dose of catalpol and metformin demonstrated the highest disposal slope and lowest AUC in ITT. ITT and HOMA_IR represent the index of insulin sensitivity where HOMA-IR mostly predicts the hepatic insulin sensitivity [34], which does not necessarily reflect the peripheral insulin sensitivity [35]. While *ITT* represents whole body insulin action or sensitivity [30]. Hence, these two parameters represent different aspects of insulin resistance although the association between insulin resistance obtained from the HOMA and ITT is significant [36]. In both HOMA_IR and ITT, a high dose of catalpol seems to be effective in ameliorating insulin sensitivity.

Our findings are in agreement with other researchers where catalpol’s effect in controlling the glycaemic parameters was reported. Li et al. and Xu et al., reported that catalpol produced a dose-dependent reduction in fasting plasma glucose, total cholesterol (TC) and triglyceride (TG) level in HFD/STZ-induced diabetic mice [16,18]. Another study in db/db mice, catalpol (40–160 mg/kg p.o. for 4 weeks) significantly reduced FBG, TC, TG, and glycated serum protein concentrations with an improvement in glucose tolerance and reduced insulin resistance [20]. Mice fed with a HFD and catalpol (100 mg/kg, p.o. for four weeks) reduced insulin resistance as evidenced by the reductions in FBG, plasma insulin concentration and increased responsiveness to injected insulin [21]. Catalpol (5–50 mg/kg, i.v. daily for two weeks) produced a dose-dependent reduction in FBG in a rat model of STZ/high fat/high sugar-induced T2DM [37]. These findings suggested the dose-dependent reduction in FBG and other parameters by catalpol with the minimum per-oral dose started at 40 mg/kg. However, in our study, we have reported that catalpol at 100 mg/kg and 200 mg/kg can reduce the FBG dose-dependently but other glycaemic parameters have no significant response at low dose, i.e., 100 mg/kg, although it exhibited some reduction.

In our study, we have investigated the possible molecular mechanism of catalpol which is responsible for improving glucose homeostasis and insulin sensitivity in diabetic mice. In the previous study, we reported that catalpol (200 mg/kg, p.o.) increased the PPAR-γ gene and protein expression, glucokinase gene expression, and increased glycogen content in the liver, which correlates to glycaemic control and insulin sensitivity in the liver [22]. In the current study, we have investigated the mRNA levels of insulin receptor substrate 1 (IRS-1), PI3K, Akt-2, and GLUT-4 and GLUT4 protein in skeletal muscle of normal control, diabetes control and treatment groups. Insulin signaling is the key to glucose uptake into the insulin sensitive tissues for their utilization as energy. Insulin binds to the α subunits of the insulin receptor (IR) and activates the tyrosine kinase in the β subunit. Once tyrosine kinase of the β subunit is activated, it promotes the threonine phosphorylation of IRS-1 and in turn stimulates the activation of the PI3K/Akt pathway which further translocates the GLUT4 onto the plasma membrane. GLUT4 is the transporter responsible to transport glucose from extracellular into intracellular. Once the glucose is transported into the skeletal muscle, it is utilized to generate ATP as the energy [38]. In skeletal muscle, this PI3K/Akt activation is an essential step for insulin-induced GLUT4 translocation, leading to glucose uptake [39]. In insulin resistance and T2DM, insulin signaling is disrupted resulting in a downregulation of GLUT4 translocation on the surface of the muscle membrane. In HFD/STZ model, HFD feed to animals results in insulin resistance [27,28]. In the present investigation, IRS-1, PI3k, Akt-2, and GLUT4 gene and GLUT4 protein expression in skeletal muscle of diabetic mice is significantly low as compared to normal control mice and catalpol significantly upregulated these genes and GLUT4 protein in skeletal muscle. Similar changes in the protein levels of IRS-1, p-IRS-1, PI3K, Akt, p-Akt, and GLUT4 in the skeletal muscle of ob/ob mice [17] and HFD-induced prediabetic mice [40] by catalpol were reported. It was demonstrated that phosphorylated insulin signaling proteins and GLUT4 protein are significantly low in diabetic condition and catalpol have significantly improved these proteins in diabetic state [17,40]. Overall, it is confirmed that catalpol has the ability to change these proteins level through elevating the IRS-1, PI3k, Akt-2 and GLUT4 genes. Thus, catalpol enhances the insulin sensitivity and glucose uptake in the skeletal muscle of diabetic mice through augmenting the insulin signaling pathway.

Further, the hypoglycemic effect of catalpol was suggested to be mediated through improved muscle mitochondrial function [16] and mitochondrial biogenesis [18]. Catalpol has demonstrated increased expression of PGC-1α mRNA [16] and activated AMPK/PGC-1α/TFAM signaling in skeletal muscle. Antidiabetic activity of catalpol is linked to the increased glucose uptake and ATP production in skeletal muscle and augmentation of insulin sensitivity [18]. Insulin is a key regulator of mitochondrial function and several studies in humans demonstrated the direct effects of insulin on the mitochondrial function whereby insulin infusion leads to an augmented expression of mitochondrial proteins, increased oxidative enzyme activity, and elevated ATP synthesis in muscle [41,42]. The direct effect of insulin on mitochondrial function is attenuated in insulin resistance subjects [41] and HFD fed animals [43]. These studies support the insulin directed mitochondrial biogenesis and oxidative capacity whereas, insulin resistance contributes to mitochondrial dysfunction. Other studies also support this hypothesis where genetic induction of insulin resistance through the deletion of IRS genes has a dramatic effect on mitochondrial function thus insulin resistance leads to the development of mitochondrial dysfunction [44,45]. Mitochondria abundantly present in the skeletal muscle and mitochondrial dysfunction is correlated with insulin resistance in skeletal muscle [46]. Indeed, skeletal muscle insulin resistance in a HFD model disrupts mitochondrial biogenesis and oxidative capacity [47]. There is definitely a clear link established between insulin and mitochondrial function, however it is difficult to explain which pathway regulates the other. There are studies that support both the possibility i.e., insulin resistance initiate mitochondria dysfunction [39] and mitochondria dysfunction initiates insulin resistance [47]. Catalpol has demonstrated a significant improvement in the expression of insulin signaling genes, leading to improved insulin sensitivity, and that could have a link with the enhanced mitochondrial function. To support this hypothesis, we investigated the gene/protein expression that regulates mitochondrial biogenesis/function and measured the mitochondrial function (state-3/stae-4 respiration) and CS activity in the skeletal muscle.

PGC-1α is the master regulator of mitochondrial biogenesis [48] and its expression increased due to cellular ATP demand (exercise, cold exposure, and fasting) [48,49,50,51]. PGC-1α is a coactivator of nuclear respiratory factor-1 (NRF-1) and PPAR-γ and α [52,53,54]. The expression of many mitochondrial genes, including OXPHOS genes and mitochondrial transcription factor A (TFAM), is regulated by NRF-1, which is crucial for replication of the mitochondrial genome [54]. PGC-1α and NRF-1 expression are reduced in diabetic and insulin-resistant human subjects [55]. Thus, insulin-resistant subjects have fewer mitochondria in their skeletal muscle, possibly due to decreased PGC-1α and β expression [54,56]. The expression of PGC-1α is regulated by different pathways. One such pathway is through activation of endothelial nitric oxide synthase (eNOS)/NO/cGMP/PGC-1α axis and eNOS plays an important role in mitochondria biogenesis through PGC-1α [57,58]. SIRT1 is another important factor that regulates PGC-1α activation and interestingly, it is connected with AMPK activity. Phosphorylated AMPK enhances SIRT1 activity by increasing intracellular NAD+ levels [59]. This translates in the deacetylation of SIRT1 targets such as PGC-1α in response to the pharmacological or physiological AMPK activation [59]. AMPK and SIRT1 gene expression is significantly higher in catalpol treated mice as compared to diabetic control mice. A significant increase in phosphorylated and total AMPK protein was observed in catalpol treated groups. Hence, PGC-1α expression could be regulated due to AMPK phosphorylation and SIRT1 activation by catalpol. AMPK axis is the predominant regulatory mechanism in skeletal muscle so, catalpol is augmenting the AMPK phosphorylation mediated PGC-1α expression in skeletal muscle which is reflected as increased AMPK-SIRT1-PGC-1α gene expression in skeletal muscle. Our observation is in agreement with the reported pathway of AMPK/PGC-1α/TFAM by catalpol [18]. With this mechanism established for catalpol, it has some similarity with metformin action against type-2 diabetes where metformin, an AMPK activator, increases PGC-1α expression [60]. However, we further investigated to understand the insulin sensitizing effect of catalpol by measuring PPAR-γ protein expression in skeletal muscle. PPAR-γ expression in normal and diabetic animals did not show any significant difference. This observation is in agreement with other researchers who reported similar findings in humans with no difference in the expression of PPAR-γ in the skeletal muscle of normal and diabetic patients [61]. However, catalpol treatment significantly increased the PPAR-γ protein expression in skeletal muscle. Metformin didn’t show any significant change in PPAR-γ protein level versus diabetic control. PGC-1α activation leads to co-activation of NRF- 1 and 2, estrogen-related receptors (ERRs), and PPARs. NRF activation induces TFAM transcription which facilitates mtDNA synthesis leading to mitochondrial biogenesis. ERR activation increase mtDNA synthesis and improves glucose utilization, whereas PPARs activation upregulates the enzyme systems that facilitate beta-oxidation [62].

To further support the AMPK/SIRT1/PGC-1α/PPAR-γ pathway involved in skeletal muscle, we have measured the mitochondrial respiration and CS activity in all groups. The link between PGC-1α and mitochondrial respiration was demonstrated by Pagel-Langenickel et al. PGC-1α knockdown led to a significant reduction of mitochondrial-encoded cytochrome *c* oxidase subunit I gene, OCR and Akt phosphorylation [63]. The role of PGC-1α in both mitochondrial biogenesis and insulin signal transduction is well supported. We have measured the oxygen consumption rate of freshly isolated mitochondria from skeletal muscle of mice by using a commercially available kit. State-4 respiration and state-3 respiration are defined as oxygen consumption by isolated mitochondria in the absence and presence of ADP respectively on a particular substrate [64]. The substrate used in this experiment is succinate. The isolated mitochondria from the skeletal muscle of normal control animals showed a steep increase in the slope of state 3 respiration suggesting the prompt usage of ADP in ATP formation. This indicates the normal mitochondrial function without any mitochondrial injury in normal control. In contrast, the slope of state 3 respiration in diabetic control groups is markedly low, indicating a significant reduction in OCR and RCR. Our result is in agreement with other published data where it was reported that Wistar rats fed with an HFD displayed decreased mitochondrial respiration and ATP production in the soleus muscle [65]. Further, reduced state 3 respiration was also reported in type-2 diabetes patients [64]. The slope of state 3 respiration in catalpol treated groups showed marked improvement with a significant increase in OCR and RCR thus, catalpol has rescued the mitochondrial respiration in diabetic mice. However, a similar effect was not observed in metformin, indicating that its role in mitochondrial respiration is absent. Previously, it was reported that metformin has no effect OCR regardless of the respiratory states (3, 4, and uncoupled) [66]. In addition, CS activity was measured in the skeletal muscle of all animals. CS is a key mitochondrial enzyme that has been used as a biomarker for mitochondrial content and function. Low CS activity and reduced palmitate oxidation were observed in muscle cell cultures of diabetic patients as compared to healthy subjects [67,68]. Diabetic animals showed a significant reduction in CS activity with low slope value which supports the previous findings. Catalpol treatment significantly ameliorated the CS activity with an 82% increase in CS activity against diabetic control, whereas metformin did not show any significant change in CS activity, although it improved with 41% against diabetic control. The data of OCR and CS activity are well correlated in this study for all the groups. The increase in OCR and CS activity by catalpol is corroborated with the molecular pathway involving AMPK/SIRT1/PGC-1α/PPAR-γ where enzyme availability is increased for beta-oxidation in the mitochondria.

## 5. Conclusions

**Figure 7:**
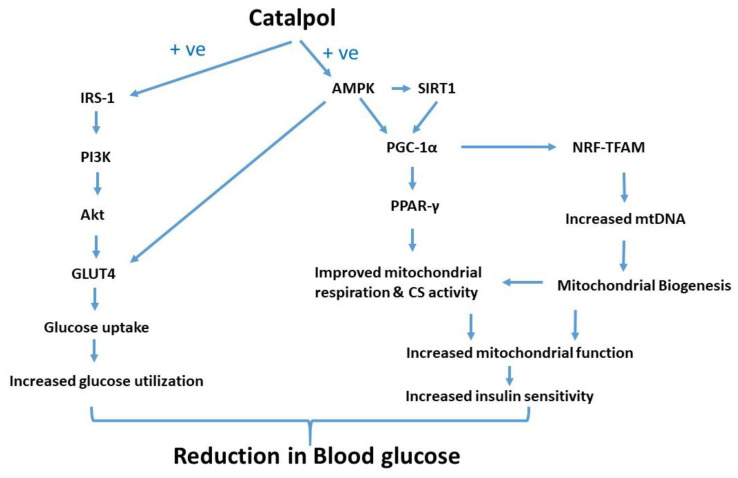
Putative molecular pathway of catalpol in the skeletal muscle of type-2 diabetic mice in amelioration of glucose homeostasis and insulin sensitivity.

Catalpol treatment to the diabetic mice significantly attenuated the diminished insulin signaling gene expression, mitochondrial respiration and CS activity. Thus, the mitochondrial function is profoundly increased by catalpol which could be mediated through augmentation of insulin signaling and/or AMPK/SIRT1/PGC-1α/PPAR-γ pathway. In contrast, metformin significantly increased p-AMPK and GLUT4 expression without any effect on mitochondrial function in the skeletal muscle. The putative molecular pathway of catalpol is depicted in Figure 7. Based on the observation, catalpol exhibits a potential antidiabetic effect by ameliorating insulin sensitivity, glucose uptake, and mitochondrial function, which needs to be further investigated in a human population.
